# Somtimes: self organizing maps for time series clustering and its application to serious illness conversations

**DOI:** 10.1007/s10618-023-00979-9

**Published:** 2023-10-20

**Authors:** Ali Javed, Donna M. Rizzo, Byung Suk Lee, Robert Gramling

**Affiliations:** 1 Department of Medicine, Stanford University, 300 Pasteur Dr, Stanford, CA 94305 USA; 2 Department of Civil and Environmental Engineering, University of Vermont, Burlington, VT USA; 3 Department of Computer Science, University of Vermont, Burlington, VT USA; 4 Department of Family Medicine, University of Vermont, Burlington, VT USA

**Keywords:** Time series clustering, Self-organizing maps, Dynamic time warping, Clustering, Serious illness conversations

## Abstract

There is demand for scalable algorithms capable of clustering and analyzing large time series data. The Kohonen self-organizing map (SOM) is an unsupervised artificial neural network for clustering, visualizing, and reducing the dimensionality of complex data. Like all clustering methods, it requires a measure of similarity between input data (in this work time series). Dynamic time warping (DTW) is one such measure, and a top performer that accommodates distortions when aligning time series. Despite its popularity in clustering, DTW is limited in practice because the runtime complexity is quadratic with the length of the time series. To address this, we present a new a self-organizing map for clustering TIME Series, called SOMTimeS, which uses DTW as the distance measure. The method has similar accuracy compared with other DTW-based clustering algorithms, yet scales better and runs faster. The computational performance stems from the pruning of unnecessary DTW computations during the SOM’s training phase. For comparison, we implement a similar pruning strategy for K-means, and call the latter K-TimeS. SOMTimeS and K-TimeS pruned 43% and 50% of the total DTW computations, respectively. Pruning effectiveness, accuracy, execution time and scalability are evaluated using 112 benchmark time series datasets from the UC Riverside classification archive, and show that for similar accuracy, a 1.8$\times$ speed-up on average for SOMTimeS and K-TimeS, respectively with that rates vary between 1$\times$ and 18$\times$ depending on the dataset. We also apply SOMTimeS to a healthcare study of patient-clinician serious illness conversations to demonstrate the algorithm’s utility with complex, temporally sequenced natural language.

**Supplementary Information:**

The online version contains supplementary material available at 10.1007/s10618-023-00979-9.

## Introduction

By 2025, it is estimated that more than four hundred and fifty exabytes of data will be collected and stored daily (WorldEconomicForum 2019). Much of that data will be collected continuously and represent phenomena that change over time. We propose that fully understanding the meaning of these data will require methods capable of efficiently visualizing and analyzing large amounts of time series. Examples include data mined by the Internet of Things (CRS 2020; Evans 2011), lexicon and natural language (e.g., Bentley et al. 2018; Ross et al. 2020; Reagan et al. 2016; Chu et al. 2017), biomonitors (Gharehbaghi and Lindén 2018), streamflow, barometric pressure and other environmental sensor data (e.g., Hamami and Dahlan 2020; Javed et al. 2020a; Ewen 2011), social media interactions (e.g., De Bie et al. 2016; Javed and Lee 2018, 2016, 2017), and hourly financial data reported by fluctuating world stock and currency markets (Lasfer et al. 2013). In response to the increasing amounts of time-oriented data available to analysts, the applications of time-series modeling are growing rapidly (e.g., Minaudo et al. 2017; Dupas et al. 2015; Mather and Johnson 2015; Bende-Michl et al. 2013; Iorio et al. 2018; Gupta and Chatterjee 2018; Pirim et al. 2012; Souto et al. 2008; Flanagan et al. 2017).

Time series modeling is computationally “expensive” in terms of processing power and speed of analysis. Indeed, as the numbers of observations or measurement dimensions for each observation increase, the relative efficiency of time series modeling diminishes, creating an exponential deterioration in computational speed. Under conditions where computing power is in excess or when the speed for generating results is not of concern, these challenges would be less pressing. Currently, however, these conditions are rarely met, and the accelerating rate of data collection promises to outpace the computational infrastructure available to most analysts.

In this work, we embed distance-pruning into a new artificial neural network—**S**elf-**O**rganizning **M**ap for **time** **s**eries (SOMTimeS) and **K**-means for **time** **s**eries (K-TimeS) to improve the execution time of clustering methods that used Dynamic Time Warping (DTW) for large time series applications. The single layered SOMTimeS is computationally faster than the deep layered classifiers (Wang and Jiang 2021) making it suitable for time series analysis. The popular K-Means clustering of K-TimeS is fast, particularly for well-separated clusters, but lacks the outstanding visualization capabilities of SOMTimeS. The computational efficiency of these algorithms is attributed to the pruning of unnecessary DTW computations during the training phases of each algorithm. When assessed using 112 time series datasets from the University of California, Riverside (UCR) classification archive, SOMTimeS and K-TimeS prunded 43% and 50% of the DTW computations, respectively. While the pruning efficiency and resulting speed-up vary depending on the dataset being clustered, on average, there is a 1.8$\times$ speed-up over all 112 of the archived datasets. To the best of our knowledge, K-TimeS and SOMTimeS are the fastest DTW-based clustering algorithms to date.

SOMTimeS is designed for users who wish to leverage DTW (i.e., optimally align two time series) as well as the SOM’s visualization capabilities when clustering time series data of high complexity. To explore the potential utility of SOMTimeS in this regard, we show the algorithm’s ability to cluster and visualize time series data (i.e., conversational narratives) from highly emotional doctor-family-patient conversations. Understanding and improving serious illness communication is a national priority for 21st century healthcare; but, our existing methods for measuring and analyzing such data are cumbersome, human intensive, and far too slow to be relevant for large epidemiological studies, communication training, or time-sensitive reporting. Here, we use data from an existing multi-site epidemiological study of serious illness conversations as one example of how efficient computational methods can add to the science of healthcare communication.

The remainder of this paper is organized as follows. Section 2 provides background information on SOMs and DTW. Section 3 presents the SOMTimeS algorithm. Sections 4 and  5 provide the performance measures for SOMTimeS, K-TimeS, and another DTW-based clustering algorithm – TADPole using the UCR benchmark datasets, and then show proof-of-concept of the SOMTimeS algorithm as an exploratory data analysis tool involving health care conversations. Section 6 discusses the results. Section 7 concludes the paper and suggests future work.

## Background

Similar to the work of Silva and Henriques (2020), Li et al. (2020), Parshutin and Kuleshova (2008), and Somervuo and Kohonen (1999), SOMTimeS is a new artificial neural network that embeds a distance-pruning strategy into a DTW-based Kohonen self-organizing map. While the original Kohonen SOM (see details in Sect. 2.1) is linearly scalable with respect to the number of input data, it often performs hundreds of iterations (i.e., epochs) when self-organizing or clustering the training data. Each epoch requires $n \times M$ distance calculations, where *n* is the number of observations and *M* is the number of nodes in the network map. While work has been performed to improve the SOM speed using both hardware (Dias et al. 2021; de Abreu de Sousa et al. 2020) and algorithmic (Conan-Guez et al. 2006) solutions, the large number of required distance calculations is problematic, particularly when the distance measure is computationally expensive, as is the case with DTW (see Sect. 2.2).

Originally introduced in 1970 s for speech recognition (Sakoe and Chiba 1978), DTW continues to be one of the more robust, top performing, and consistently chosen learning algorithms for time series data (Xi et al. 2006; Ding et al. 2008; Paparrizos and Gravano 2016, 2017; Begum et al. 2015; Javed et al. 2020b). Its ability to shift, stretch, and squeeze portions of the time series helps address challenges inherent to time series data (e.g., optimize the alignment of two temporal sequences). Unfortunately, the ability to align the temporal dimension comes with increased computational overhead that has hindered its use in practical applications involving large datasets or long time series clustering (Javed et al. 2020b; Zhu et al. 2012). The first subquadratic-time algorithm ($O(m^2/\log \log {m})$) for DTW computation was proposed by Gold and Sharir (2018), which for comparison is still more computationally expensive than the simpler Euclidean distance (*O*(*m*)).

To address the computational cost, several studies have presented approximate solutions (Zhu et al. 2012; Salvador and Chan 2007; Al-Naymat et al. 2009). To the best of our knowledge, TADPole by Begum et al. (2015) is the only clustering algorithm (see supplementary material Section 8.1) that speeds up the DTW computation without using an approximation. It does so by using a bounding mechanism to prune the expensive DTW calculations. Yet, when coupled with the clustering algorithm (i.e., Density Peaks of Rodriguez and Laio 2014), it still scales quadratically. Thus, even after decades of research (Zhu et al. 2012; Begum et al. 2015; Lou et al. 2015; Salvador and Chan 2007; Wu and Keogh 2020), the almost quadratic time complexity of DTW-based clustering still poses a challenge when clustering time series in practice.

### Self organizing maps

**Fig. 1:**
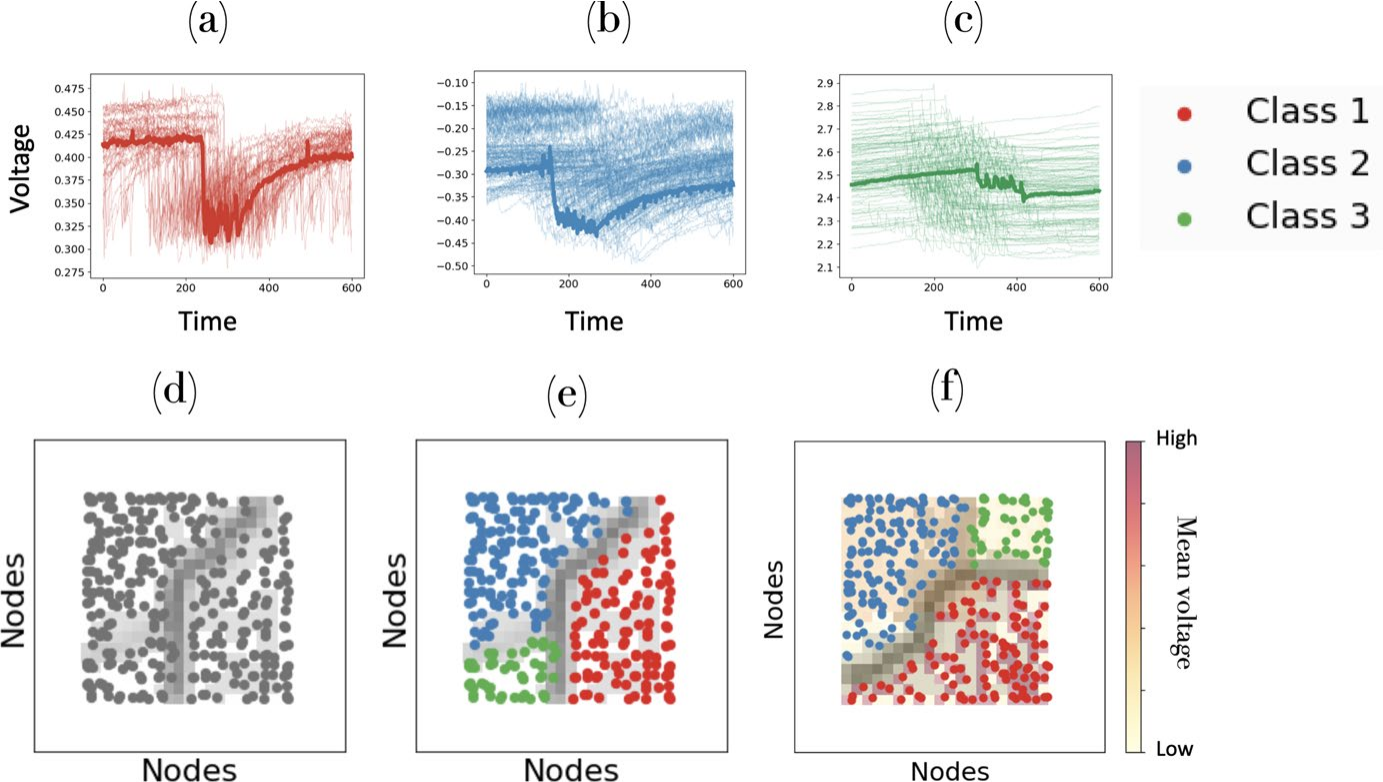
Results of clustering times series from one of the UCR archive datasets—InsectEPGRegularTrain, using a Self-Organized Map with DTW as the distance measure (a.k.a. SOMTimeS). **a**–**c** Three sets of classified time series associated with voltage changes that capture interactions between insects and their food source (e.g., plants). In each panel a single observation (time series) is highlighted. **d** Unified distance matrix (gray shading) that separates the clustered observations on the 2D grid, **e** color-codes the observations by cluster ID, and **f** superimposes the mean voltage value in the background

The Kohonen self-organizing map (Kohonen et al. 2001; Kohonen 2013) may be used to either cluster or classify observations, and has advantages when visualizing complex, nonlinear data (Alvarez-Guerra et al. 2008; Eshghi et al. 2011) Additionally, it has been shown to outperform other parametric methods on data with outliers or high variance (Mangiameli et al. 1996). Similar to methods such as logistic regression and principal component analysis, SOMs may be used for feature selection, as well as mapping input data from a high-dimensional space to a lower-dimensional (typically two-dimensional) space/map. To demonstrate the utility of 2-D visualization, we used the DTW-based SOM to cluster data from one of the UCR archive datasets (InsectEPGRegularTrain) onto a 2-D mesh (see Fig. 1). The input time series data represent voltage changes from an electrical circuit designed to capture the interaction between insects and their food source (e.g., plants). While these data had already been classified into three categories, we show the results from the DTW-based SOM clustering in Fig. 1a–c. The self-organized time series may also be plotted in 2-D space (see map of Fig. 1d); each gray dot represents a input time series. The gray shading represents what is known as a unified distance matrix or U-matrix (Ultsch 1993), and is obtained by calculating the average difference between the weights of adjacent nodes in the trained SOM. These differences are plotted in a gray scale on the trained 2-D mesh. Darker shading represents higher U-matrix values (larger average distance between observations). In this manner, the U-matrix may be used to help assess the quality and the number of clusters. In Fig. 1e, we color-code the three clustered observations; labels (should they exist) could also be superimposed. Finally, any information (i.e, input features or metadata) associated with the observations may also be visualized/superimposed in the same 2-D space. The latter allows users or domain experts to explore associations and the importance of individual input features with the self-clustered results. As an example we visualize the mean voltage value in the background in Fig. 1f. The ability to visualize individual input features in the same space as the clustered observations (known as component planes) makes the SOM a powerful tool for exploratory data analysis and feature selection.

### Dynamic time warping

**Fig. 2:**
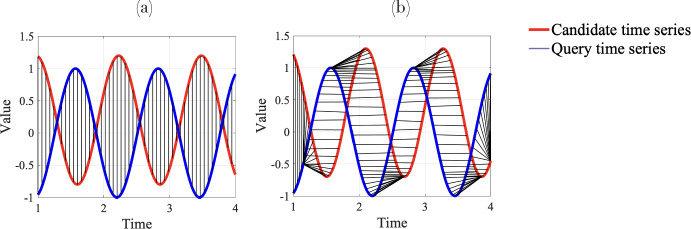
Alignment between two times series for calculating **a** Euclidean distance and **b** DTW distance

DTW is recognized as one of the most accurate similarity measures for time series data (Paparrizos and Gravano 2017; Rakthanmanon et al. 2012; Johnpaul et al. 2020; Javed et al. 2020b). While the most common measure, Euclidean distance, uses a one-to-one alignment between two time series (e.g., labeled candidate and query in Fig. 2a), DTW employs a one-to-many alignment that warps the time dimension (see Fig. 2b) in order to minimize the sum of distances between time series samples. As such, DTW can optimize alignment both globally (by shifting the entire time series left or right) and locally (by stretching or squeezing portions of the time series). The optimal alignment should adhere to three rules:

1. Each point in the query time series must be aligned with one or more points from candidate time series, and vice versa.
2. The first and last points of the query and a candidate time series must align with each other.
3. No cross-alignment is allowed; that is, the aligned time series indices must increase monotonically.

DTW is often restricted to aligning points only within a moving window of a fixed size to improve accuracy and reduce computational cost. The window size may be optimized using supervised learning on the training data. When supervised learning is not possible (i.e., clustering), a window size amounting to 10% of the observation data is usually considered adequate (Ratanamahatana and Keogh 2004). We fixed the DTW window constraint at 5% of the length of the observation data as it was shown to be the optimal window size for the UCR archive Paparrizos and Gravano (2016, 2017).

#### Upper and lower bounds for DTW-based distance

**Fig. 3:**
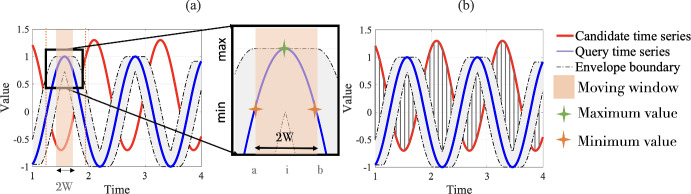
Two steps of calculating the LB_Keogh tight lower bound for DTW in linear time: **a** determine the envelope around a query time series, and **b** sum the point to point distance shown in grey lines between the envelope and a candidate time series as LB_Keogh (Eq. 2)

SOMTimeS uses distance bounding to prune the DTW calculations performed during the SOM unsupervised learning. This distance bounding involves finding a tight upper and lower bound. Because DTW is designed to find a mapping that minimizes the sum of the point-to-point distances between two time series, that mapping can never result in a summed distance that is greater than the sum of point-to-point Euclidean distance. Hence, finding the tight upper bound is straight forward—it is the Euclidean distance (Keogh 2002). To find the lower bound, we use a method—the LB_Keogh method (Keogh 2003), common in similarity searches (Keogh 2003; Ratanamahatana et al. 2005; Wei et al. 2005) and clustering (Begum et al. 2015). The LB_Keogh method comprises two steps (see Fig. 3a, b). Given a fixed DTW window size, *W*, one of the two time series (called the query time series, Q) is bounded by an envelope having an upper ($U_i$) and lower boundary ($L_i$) calculated at time step *i*, respectively, as:

1 $$\begin{aligned} \begin{aligned} U_i = \max (q_{a},\ldots ,q_{i},\ldots ,q_{b}) \\ L_i = \min (q_{a},\ldots ,q_{i},\ldots ,q_{b}), \end{aligned} \end{aligned}$$

where $a = i-W$, and $b= i+W$ (see Fig. 3a). In the second step, the LB_Keogh lower bound is calculated as the sum of Euclidean distance between the candidate time series and the envelope boundaries (see vertical lines of Fig. 3b). Equation 2 shows the formula for calculating the LB_Keogh lower bound:

2 $$\begin{aligned} \text {LB\_Keogh}= \sqrt{ \sum _{i=1}^{m}{ {\left\{ \begin{array}{ll} (t_i - U_i)^2,&{} \text {if } t_i > U_i\\ (t_i - L_i)^2, &{} \text {if } t_i < L_i\\ 0, &{} \text {otherwise} \end{array}\right. } }} \end{aligned}$$

where $t_i$, $U_i$, and $L_i$ are the values of a candidate time series, the upper and lower envelope boundary, respectively, at time step *i*.

#### The UCR archive

The UCR time series classification archive (Dau et al. 2018), with thousands of citations and downloads, is arguably the most popular archive for benchmarking time series clustering algorithms. The archive was born out of frustration, with studies on clustering and classification reporting error rates on a single time series dataset, and then implying that the results would generalize to other datasets. At the time of this writing, the archive has 128 datasets comprising a variety of synthetic, real, raw and pre-processed time series data, and has been used extensively for benchmarking the performance of clustering algorithms (e.g., Paparrizos and Gravano 2016, 2017; Begum et al. 2015; Javed et al. 2020b; Zhu et al. 2012). We excluded sixteen of the archive datasets because they contained only a single cluster, or had time series lengths that vary, and the latter prohibited a fair comparison of SOMTimeS to K-TimeS. Thus, 112 datasets are used to evaluate the accuracy, execution time, and scalability of SOMTimeS.

## The SOMTimeS algorithm

**Fig. 4:**
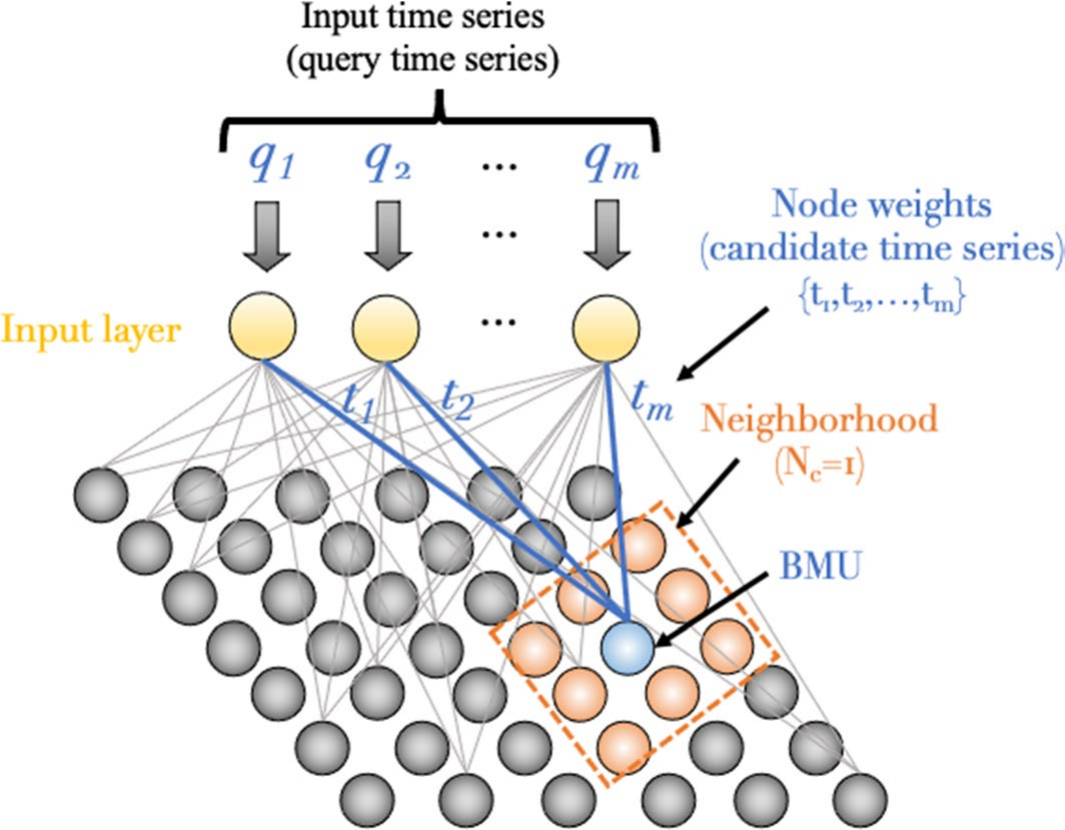
Schematic of the Kohonen self-organizing map (after Kohnen, 2001) showing weights (candidate time series) of the Best Matching Unit (BMU) in blue surrounded by a user-specified neighborhood ($N_c$)

SOMTimeS (see Pseudocode 1) is a variant of the SOM, where each input observation (i.e., query time series) is compared with the weights (i.e., candidate time series) associated with each node in the 2-D SOM mesh (see Fig. 4). During training, the comparison (or distance calculation) between these two time series is performed to identify the SOM node whose weights are most similar to a given input time series; this node is identified as the “Best Matching Unit (BMU)”. Once the nodal weights (candidate time series) of the BMU have been identified, these weights (and those of the neighborhood nodes) are updated to more closely match the query time series (Line 17 of Pseudocode 1). This same process is performed for all query time series in the dataset—defined as one iteration. While iterating through some user-defined fixed number of iterations, both the neighborhood size and the magnitude of change to nodal weights are incrementally reduced. This allows the SOM to converge to a solution (stable map of clustered nodes), where the set of weights associated with these self-organized nodes now approximate the input time series (i.e., observed data). In SOMTimeS, the distance calculation is done using DTW with bounding, which helps prune the number of DTW calculations required to identify the BMU.

**Fig. 5:**
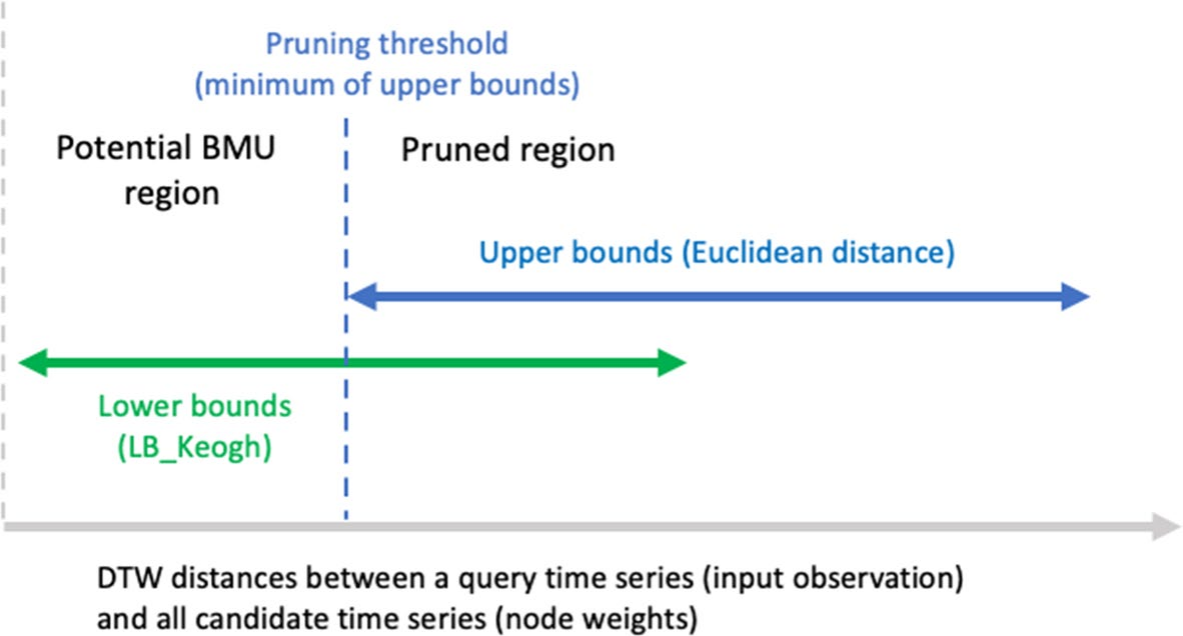
Identification of a qualification region in SOMTimeS

### Pruning of DTW computations

Pruning is performed in two steps. First, an upper bound (i.e., Euclidean distance) is calculated between the input observation and each weight vector associated with the SOM nodes (Line 9 of Pseudocode 1). The minimum of these upper bounds is set as the pruning threshold (see dotted line in Fig. 5). Next, for each SOM node, we calculate a lower bound (i.e., LB_Keogh; see Line 10). If the calculated lower bound is greater than the pruning threshold, that respective node is pruned from being the BMU. If the lower bound is less than the pruning threshold, then that SOM node lies in what we refer to as the potential BMU region (see Fig. 5, and Line 12). As a result, the more expensive DTW calculations are performed only for the nodes in this potential BMU region. The node having the minimum summed distance is the BMU.

After identifying the BMUs for each input time series, the BMU weights, as well as the weights of nodes in some neighborhood of the BMUs, are updated to more closely match the respective input time series using a traditional learning algorithm based on gradient descent (Line 17 of Pseudocode 1). Both the learning rate and the neighborhood size are reduced (see lines 19 and 20) over each epoch until the nodes have self-organized (i.e., algorithm has converged). In this work, unless otherwise stated, SOMTimeS is trained for 100 epochs. To further reduce the SOM execution time, the set of input (i.e., query) time series may be partitioned in a manner similar to Wu et al. (1991), Obermayer et al. (1990) and Lawrence et al. (1999) for parallel processing (see Line 5). We should also note that after convergence, SOMTimeS may be used to *classify* observations into a given number of clusters should a known number of classes exist. This is done by setting the mesh size equal to *k* (i.e., desired number of classes), and using the weights of the BMUs for direct class assignment. Python implementation of SOMTimeS is available at Python Package Index (Javed 2021a) and Github (Javed 2021b).
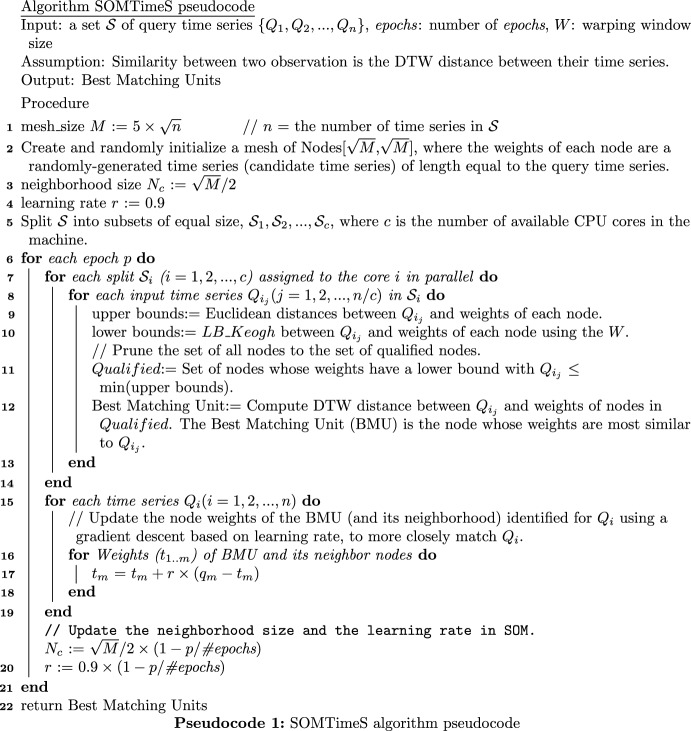


### K-TimeS: generalization of the pruning strategy to K-means

For a more equitable comparison of the speed-up and clustering quality, we embed DTW with a similar pruning strategy into a K-means clustering algorithm, and call this algorithm—K-TimeS. Given some desired number of classes, *k*, training comprises two phases. In phase one, centroids are calculated, and in the phase two, the observations (i.e., query time series) are assigned to their closest centroid using DTW as the distance measure. This continues until some termination condition is met (e.g., number of iterations or convergence). The DTW distance bounding is used in the second phase to prune unnecessary DTW computations required in identifying the closest centriod. Specifically, we assign a centroid to the pruned region of Fig. 5 when it’s lower bound (i.e., $LB\_Keogh$) distance to a given query time series is greater than the minimum of the upper bound (i.e., Euclidean) distances between the *k* centroids and the query time series (see pruning threshold in Fig. 5). The pruned centroid cannot be the closest centroid to the given query time series, since there exists at least one other centroid whose DTW distance to the query time series is less than or equal to the pruned centroid’s. The computational speed-up achieved in the K-TimeS algorithm results from avoiding the expensive DTW computation between the pruned centroid and the query time series.

## Performance evaluations

The performance of SOMTimeS may be quantified in three important ways: (1) clustering accuracy (Sect. 4.1), (2) pruning speedup (Sects. 4.2), and (3) scalability (Sect. 4.3). Accuracy is reported using the six assessment metrics of Table 1. These include the Adjusted Rand Index (ARI) (Santos and Embrechts 2009), Adjusted Mutual Information (AMI) (Romano et al. 2016), the Rand Index (RI) (Hubert and Arabie 1985), Homogeneity (Rosenberg and Hirschberg 2007), Completeness (Rosenberg and Hirschberg 2007), and Fowlkes Mallows index (FMS) (Fowlkes and Mallows 1983). We report the speed-up achieved for SOMTimeS and K-TimeS using the 112 UCR datasets. For scalability, we report the number of DTW computations and execution time as a function of *problem size*, defined as $\sum _{i=1}^{n}{|Q|_i}$, where |*Q*| is the length of times series *Q*, and *n* is the total number of time series in the dataset. The presence of a few large datasets in the archive makes it more informative to visualize problem size as the natural logarithm (see Figure S1 in Supplementary Material). Additionally, we report clustering accuracy in terms of ARI for the original Kohonen self-organizing map (i.e., using Euclidean distance); see Supplemental Material Table S1.

### Clustering accuracy

**Table 1:** Clustering performance shown for K-TimeS, SOMTimeS, TADPole, and their respective non DTW-distance pruning counterparts using six assessment indices

Algorithm	ARI^a^		AMI^b^		RI^c^		H^d^		C^e^		FMS^f^	
	Avg	Std	Avg	Std	Avg	Std	Avg	Std	Avg	Std	Avg	Std
K-TimeS—10-iterations (with and without pruning)	0.22	0.23	0.28	0.26	0.66	0.22	0.29	0.26	0.40	0.32	0.49	0.19
SOMTimeS—10-iterations (with and without pruning)	0.21	0.23	0.27	0.24	0.70	0.15	0.28	0.24	0.31	0.26	0.47	0.20
SOMTimeS—100-iterations (with and without pruning)	0.24	0.23	0.30	0.26	0.71	0.16	0.31	0.25	0.35	0.28	0.50	0.19
TADPole—non-iterative (with and without pruning)	0.16	0.25	0.24	0.27	0.62	0.18	0.25	0.26	0.36	0.31	0.51	0.20

Values represent averages over the 112 datasets in the UCR archive; indices closer to 1 represent better performance

*All algorithms were executed on same computational machine—dual 20-Core Intel Xeon E5-2698 v4 2.2 GHz machine with 512 GB 2,133 MHz DDR4 RDIMM

^a^Adjusted Rand Index (Santos and Embrechts 2009)

^b^Adjusted Mutual Information (Romano et al. 2016)

^c^Rand Index (Hubert and Arabie 1985)

^d^Homogeneity (Rosenberg and Hirschberg 2007)

^e^Completeness (Rosenberg and Hirschberg 2007)

^f^Fowlkes Mallows index (Fowlkes and Mallows 1983)

The use of DTW distance bounding during pruning does not effect the clustering accuracy (see values of the assessment indices in Table 1). When the number of iterations (i.e., epochs) through the dataset are fixed at 10, the assessment indices for SOMTimeS and K-TimeS algorithms are comparable for the 112 datasets from the UCR archive. In practice, however, the SOM typically requires more passes through the dataset than K-means to achieve optimal accuracy. Thus, when 100 epochs are used, the SOMTimeS achieves a higher accuracy than K-TimeS for 5 of the 6 measures. Density Peaks, on the other hand, has lower average values for all six assessment indices.

**Fig. 6:**
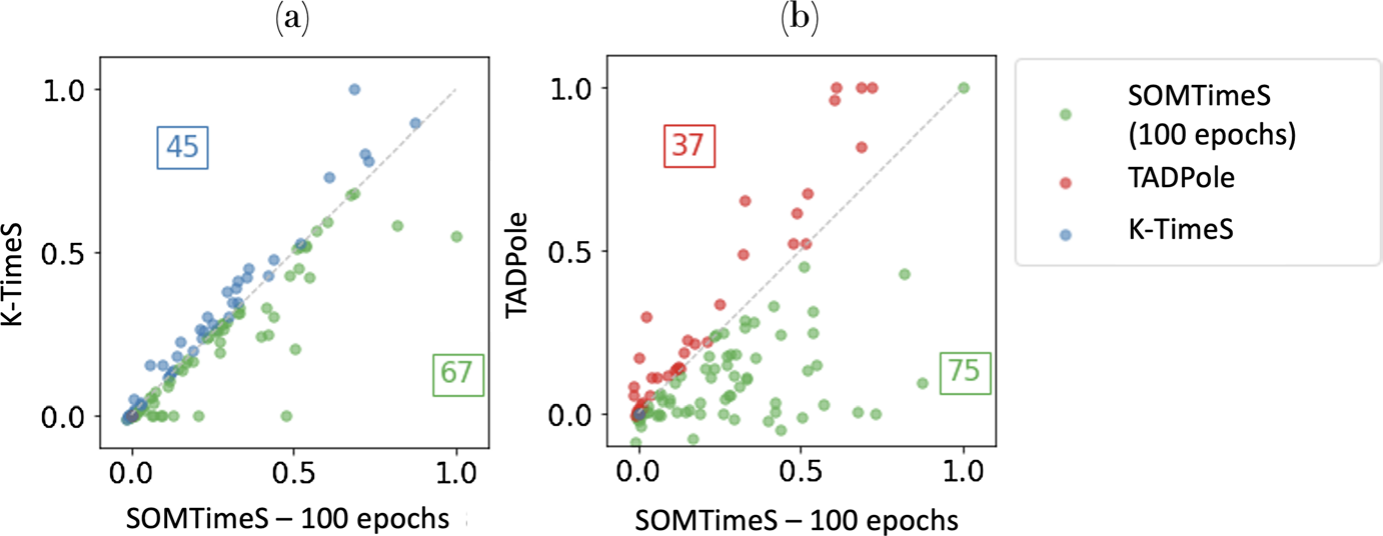
ARI scores for the SOMTimeS (shown in green) vs. **a** K-TimeS (blue), and **b** TADPole (red) across all 112 of the UCR datasets (Color figure online)

Because the performance (i.e., accuracy measures) for a given clustering algorithm is dependent on the dataset (see Table 1), we use the ARI (Adjusted Rand Index) to assess accuracy between the three DTW-based algorithms in this work. The latter is recommended as one of the more robust measures for assessing accuracy across datasets (Milligan and Cooper 1986; Javed et al. 2020b). The ARI scores for SOMTimeS at 100 epochs is plotted against (a) K-TimeS and (b) TADPole in Fig. 6 for each of the 112 URC datasets. The ARI scores shown in green (67 of the 112 datasets) lying below the 45-degree line of panel (a) represent higher accuracy for SOMTimeS; those above the diagonal (in blue) indicate that K-TimeS outperforms SOMTimeS for 45 of the 112 datasets. Fig. 6b shows SOMTimeS has higher accuracy for 75 of the 112 datasets compared to TADpole (in green), and lower accuracy for the remaining 37 datasets (red).

### Pruning speed-up

**Table 2:** Execution time in hours for clustering 112 of the UC Riverside datasets using K-TimeS, SOMTimeS, TADPole and their respective ”no-DTW distance pruning” counterparts

Algorithm	Iterations	Execution time (h)	
		(with pruning)	(without pruning)
K-TimeS	10	6	13
SOMTimeS	10	7	14
SOMTimeS	100	58	148
TADPole	Non-iterative	1011	> 1011

**Fig. 7:**
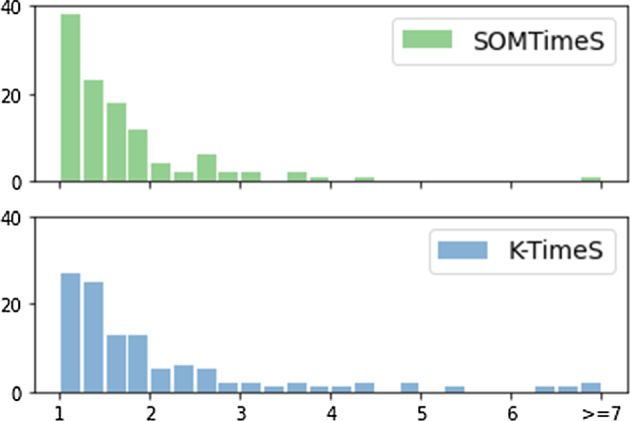
Speed-up factor achieved for the UCR archive datasets

While the speed-up times vary by dataset, the DTW distance pruning improves the execution time by a factor of 2x (on average over all 112 datasets) for both SOMTimeS and K-TimeS (see Fig. 7). K-TimeS is faster because it requires fewer iterations through the data, followed by SOMTimeS and TADPole, respectively. When the epochs are fixed at 10, both K-TimeS and SOMTimeS have comparable execution times. However, because SOMTimeS may achieve higher accuracy with more passes through the dataset (see Table 1), the execution time increases sub-linearly from $\sim 7$ to $\sim 58$ h when the number of epochs are increased from 10 to 100 (see Table 2). TADPole, with an algorithm complexity of $O(n^2)$, took 1011 h to cluster all the 112 datasets. Execution time for Density Peaks is listed as $>1011$ h.

**Total number of pruned DTW computations** K-TimeS pruned more than 50% of the DTW calculations for 34 of the datasets; whereas, SOMTimeS (with epochs set to 10 and 100, respectively) pruned more than 50% of the DTW calculations for 8 and 21 of the 112 UCR datasets, respectively. TADPole pruned more than 50% of the DTW calculations for 40 of the datasets (see Figure S2 in Supplementary Material). Despite the pruning advantage of TADPole, its quadratic complexity O($n^2$) results in more DTW computations (compared to O(*n*) in SOMTimeS), particularly for larger datasets. As a result, SOMTimeS is 17x faster when clustering the 112 datasets.

SOMTimeS is particularly well-suited to parallel execution, and when implemented in parallel, the execution time decreases as a function of available CPU. To cluster the 112 datasets in the UCR archive, SOMTimeS (at 100 epochs) took 3 h using 20 CPUs, and only 20 min when the number of SOM epochs was set to 10.

### Scalability

We studied the scalability of SOMTimeS under two difference scenarios—(1) scaling of DTW computations performed as a function of the number of input time series, and (2) change in the SOMTimeS pruning rate as a function of epochs.

**Fig. 8:**
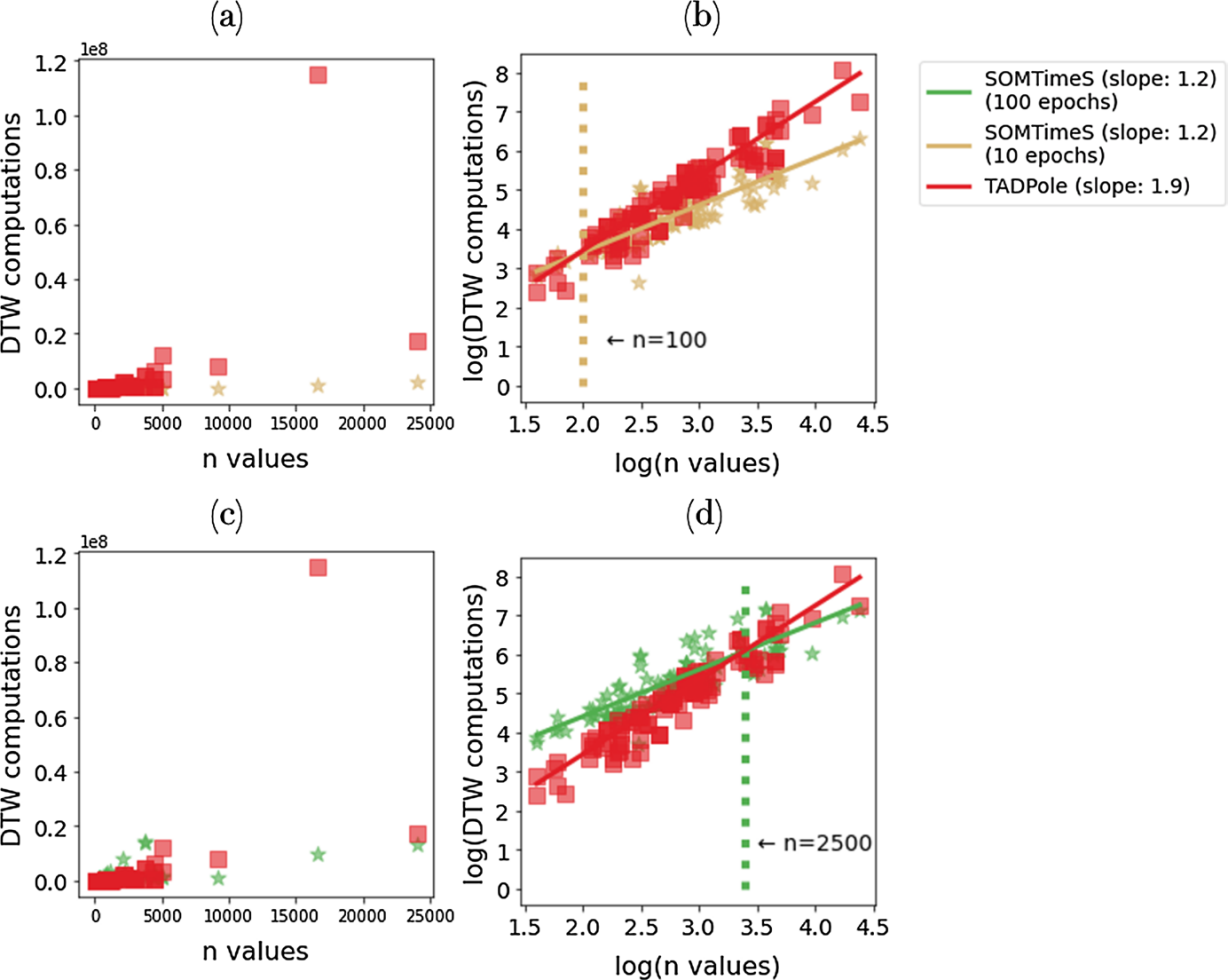
DTW computations performed as a function of dataset size using linearly scaled axes (**a**, **c**) and log-log (**b**, **d**) for TADPole (200 million DTW computations shown in red) and SOMTimeS (13 million computations at 10 epochs shown in gold; and 100 million computations at 100 epochs shown in green) (Color figure online)

**Scaling of DTW computations performed as a function of number of input time series** Because TADPole has complexity O($n^2$), the number of calls to DTW increases quadratically with the number (*n*) of input time series. As a result, there is a threshold (in terms of *n*) at which the number of calls to the DTW function is less for SOMTimeS than that of TADPole (see Fig. 8). This threshold depends on the number of training epochs, and is empirically observed to be approximately $n = 100$ and $n = 2500$, for 10 and 100 epochs, respectively. SOMTimeS and K-TimeS have similar complexities, both theoretical and empirical, when the mesh size in SOMTimeS is equal to *k*.

When clustering over all 112 of the UCR archived datasets, SOMTimeS computed the DTW measure 13 million and 100 million times (at 10 and 100 epochs), respectively. K-TimeS computed the DTW measure 8 millions times; while TADPole by comparison computed DTW 200 million times (see Fig. 8). At the dataset level, SOMTimeS had fewer calls for only 12 of the datasets (when using 10 epochs) compared to K-TimeS at 10 iterations. In comparison to TADPole, SOMTimeS had fewer calls for 88 of the datasets (when using 10 epochs), and 26 of the datasets (for 100 epochs). However, the quality of clustering for SOMTimeS at 100 epochs increases for 4 of the six assessment indices compared to K-TimeS, and TADPole.

**Fig. 9:**
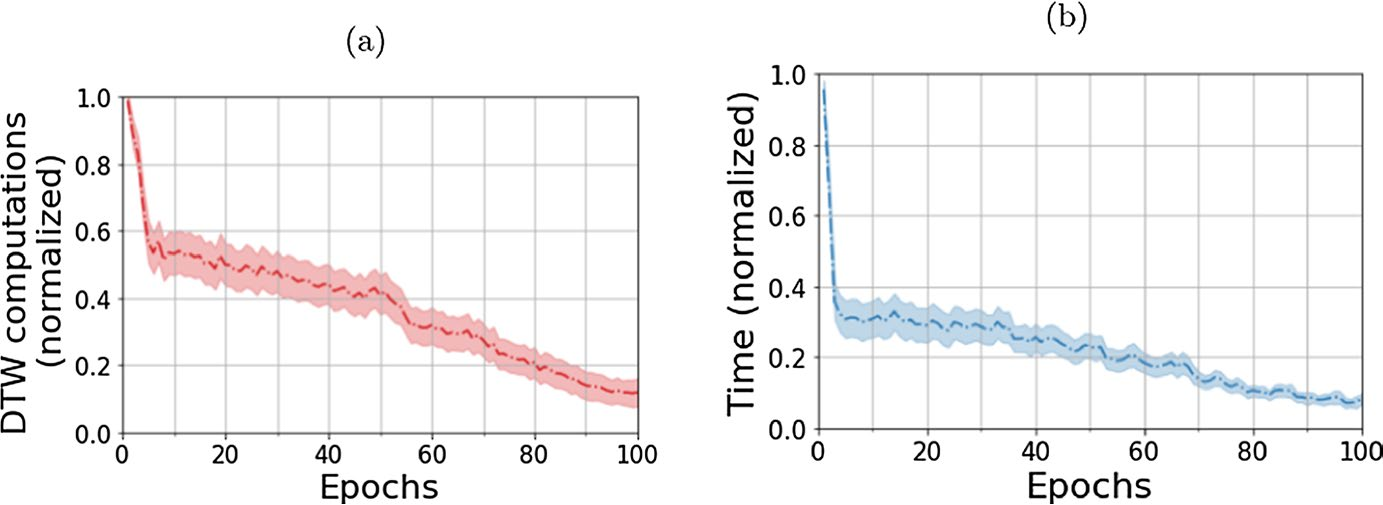
Change in the SOMTimeS pruning effect as the number of epochs increases measured as the normalized **a** number of calls to the DTW function and **b** execution time. The dashed line represents the mean value for all datasets after individually normalizing each dataset run over all epochs. The shaded region corresponds to 95% confidence interval around the mean

**Change in the SOMTimeS pruning rate as a function of epochs** When we examine the pruning effect as a function of epochs, both the number of DTW calls and the execution time decrease as the number of epochs increases. As the nodes of the SOM mesh organize, more nodes get pruned; and hence, fewer nodes exist in the unpruned region (i.e., potential BMU region of Fig. 5), which decreases the need for DTW calls. Figure 9a shows the total number of calls to the DTW function made for each dataset, normalized over all epochs. The dashed line represents the average number of calls over all datasets and the shaded region shows the 95% confidence interval. Figure 9b shows the corresponding normalized execution time. Both normalized DTW calls and execution time per epoch steadily decrease with increasing number of epochs and iterative updating of SOM weights. The elbow point, where further epochs result in a diminishing reduction of DTW calculations, is at the 6th epoch. This is called the swapover point and occurs when the self-organizing map moves from gross reorganization of the SOM weights to fine-tuning of the weights.

**Fig. 10:**
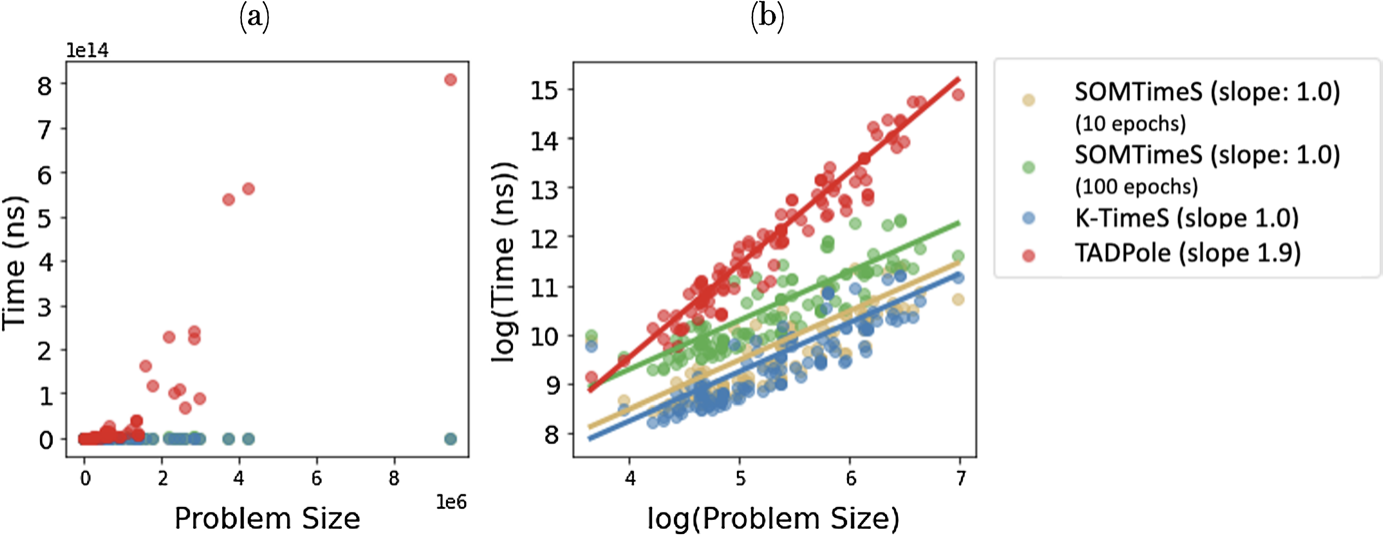
Execution time of SOMTimeS, K-TimeS, and TADPole for the 112 archived UCR datasets on a **a** linear, and **b** log scaled axes

Finally, Fig. 10 shows how SOMTimeS execution time scales with the problem size ($\sum _{i=1}^{n}{|Q|_i}$, where |*Q*| is the length of times series *Q*, and *n* is the total number of time series in the dataset). It increases at a lower rate than TADPole and is similar to K-TimeS. TADPole increases at the highest rate, consistent with its O($n^2$) complexity of DTW calculations, followed by K-TimeS with a complexity of O($n \times k \times$ number of iterations), where *k* is the number of clusters. SOMTimeS has complexity of O($n \times k \times e$), where *e* is the number of epochs. K-TimeS (at 10 iterations) is slightly faster per unit problem size than SOMTimeS (at 10 epochs) because it (1) has pruned slightly more DTW calculations, and (2) does not require weight updates.

## Application to serious illness conversations

We apply SOMTimeS to healthcare communication research in order to demonstrate the utility for identifying clinically relevant cluster analysis during complex patient-clinician interactions. We use direct observation data collected as part of the Palliative Care Communication Research Initiative (PCCRI) cohort study (Gramling et al. 2015) to demonstrate the algorithm’s utility with temporally sequenced natural language. The PCCRI is a multisite, epidemiological study that includes verbatim transcriptions of audio-recorded palliative care consultations involving 231 hospitalized people with advanced cancer, their families, and 54 palliative care clinicians. The conversations have been transcribed by human experts for natural language processing (NLP) tasks with the goal of incentivizing high-quality communication. In order to avoid sparse decile-level data in shorter conversations, we selected 171 of the conversations that were longer than 15 min in duration. The average duration of conversations was $36 \pm 16$ minutes, with an average of $327 \pm 176$ speaker turns and $4734 \pm 2280$ words. All patients completed pre- and post-conversation surveys; information gathered included age, gender, personal identity, and a short number of questions that ranged from self-rated optimism to satisfaction with the consultation.

### Discovering a clinically meaningful taxonomy of healtcare communication

Understanding and improving healthcare communication requires scalable approaches for characterizing what actually happens when patients, families, and clinicians interact in samples that are large enough to represent the diverse cultural, dialectical, decisional and clinical contexts in which these phenomena occur (Gramling et al. 2021; Tarbi et al. 2022; Clarfeld et al. 2021). Unfortunately, historical approaches to conversation analyses are too cumbersome, costly, and time-intensive to achieve this scalability, thus limiting our existing empirical knowledge about serious illness communication (Tulsky et al. 2017). Discovering and exploring clinically important patterns (i.e., clusters) of inter-personal communication amid the complex, dynamic and relational nature of clinical conversations presents a timely opportunity for scalable unsupervised machine learning methods to define a provisional taxonomy of healthcare communication. SOMTimeS is equipped to meet the need in two important ways. First, as described above, SOMTimeS offers substantial improvement in analytic efficiency necessary for identifying patterns in communication dynamics and content that unfold naturally over the temporal course of conversations. Second, as we will describe further below, the capacity for SOMTimeS to intuitively present results without pre-defining the number of clusters offers scientists of complementary disciplinary training (e.g., linguistics, anthropology, medicine, nursing, epidemiology, computer science) to empirically evaluate the number and clinical meaningfulness of communication types. Here, we use one feature of health communication (i.e., temporal ordering of conversation content) (Jaworski 2014; Labov 2013, 1980) foundational to narrative medicine (Charon 2001; Charon et al. 2016; Charon and Montello 2006, 2002).

Narrative medicine is a robust field of healthcare practice (Charon et al. 2016; Charon and Montello 2006, 2002), training and science that is grounded in the near culturally ubiquitous ways in which people explore, find and share meaning about their life experiences through stories, particularly in contexts of serious and life-threatening illness (Gramling et al. 2021; Labov 2013; Reblin et al. 2022; Barnato et al. 2016; Edlmann et al. 2019). Our previous work demonstrates that computational narrative analysis is a useful framework for characterizing the complex temporal “story arc” of naturally occurring healthcare conversations between seriously ill persons, their clinicians, and their family members (Ross et al. 2020). A central feature of narrative analysis is called temporal reference and indicates how conversation participants dynamically order the story “events” (e.g., topics, experiences, worries) happening in the past, present or future (Jaworski 2014; Labov 2013, 1980; Ross et al. 2020) Here, we demonstrate how SOMtimeS can be useful to explore clinically meaningful clusters of conversation “story arcs” using the tense of verbs and verb phrases as lexical markers of temporal reference.

### Data pre-processing: verb tense as a time series

**Fig. 11:**
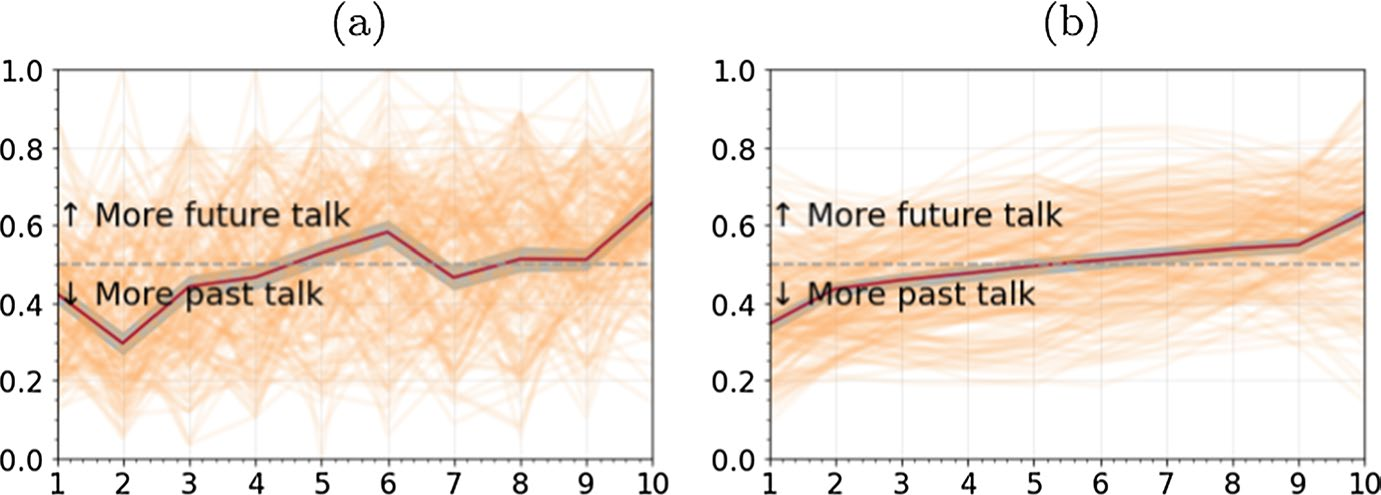
Temporal plot showing the **a** raw time series, and **b** smoothed time series for all conversations superimposed in brown; the red line represents the mean values, and the shaded region around the red lines represents 95% confidence interval (Color figure online)

We used a temporal reference tagger (Ross et al. 2020) to assign temporal reference (past, present, or future) to verbs and verb modifiers in the verbatim transcripts. Specifically, the Natural Language Toolkit (www.nltk.org) was used to classify each word in the transcripts into a part of speech and for any word classified as a verb, the preceding context is used to assign that verb (and any modifiers) to a given temporal reference. Then, each conversation was stratified into deciles of “narrative time” based on the total word count for each conversation, and a temporal reference (i.e., verb tense) time series was generated for each conversation as the proportion of all future tense verbs relative to the total number of past and future tense verbs (see Supplemental Material Figure S3a). The vertical axis in Fig. 11 represents the proportion of future vs. past talk on a per decile basis, where any value above the threshold (dashed line = 0.5) represents more future talk. Each of the 171 temporal reference time series (see Fig. 11a) were then smoothed using a 2nd-order, 9-step Savitzky-Golay filter (Savitzky and Golay 1964) (see Fig. 11b and Supplemental Material S3b). The latter smooths the time series by fitting a polynomial to data within a moving window and then uses the polynomial to replace data values. Visual inspection of the time series plots (e.g. Figure 11) showed that a DTW window size of 10% (or 1 decile) to be adequate for aligning the future vs. past talk time series.

### SOM clustering and graphical representation of temporal reference “arcs”

**Fig. 12:**
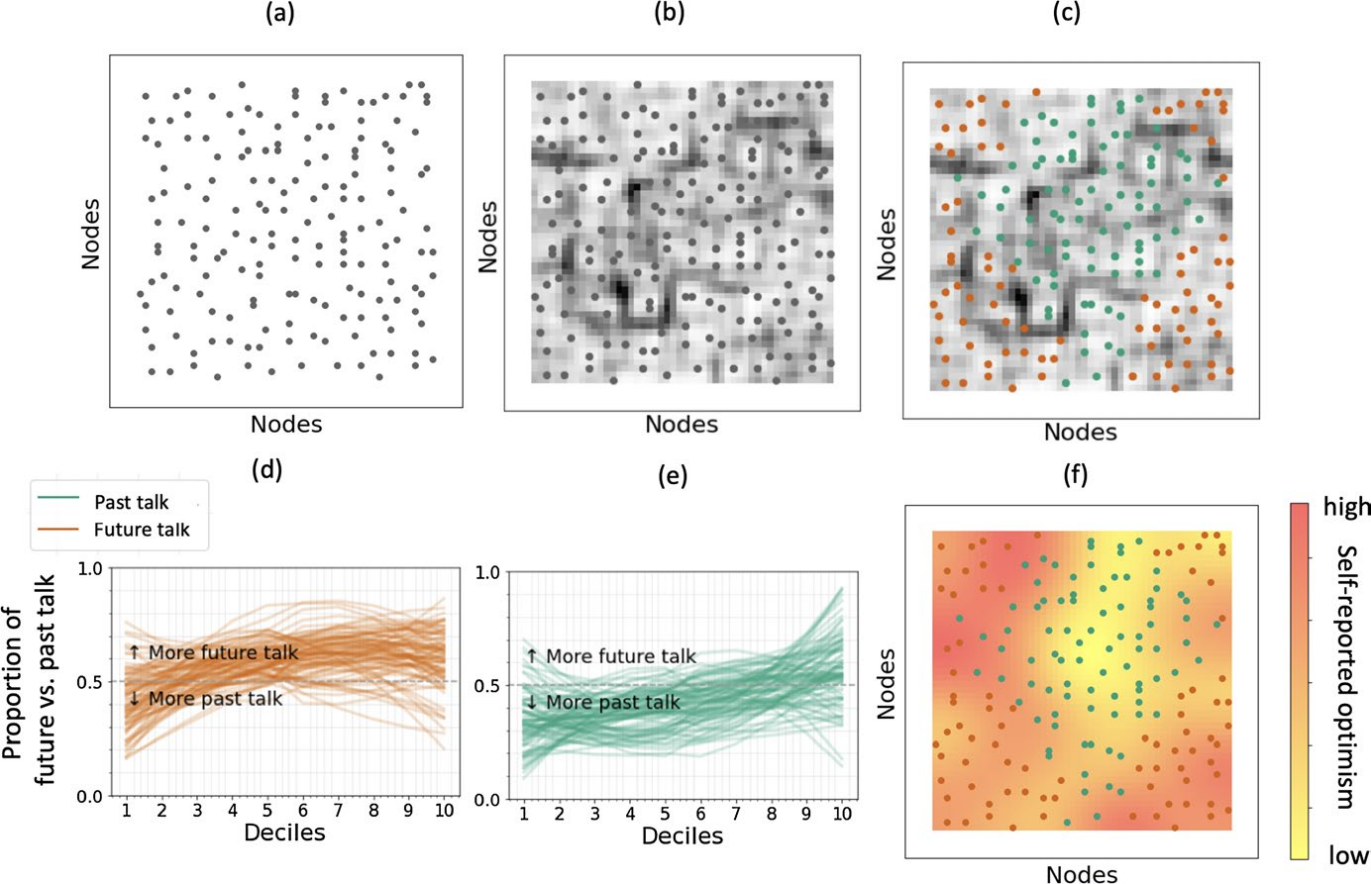
Temporal reference time series data from 171 serious illness conversations **a** self-organized on a trained 2-D map, **b** with U-matrix added, **c** color-coded into two clusters. **d**, **e** Input time series clustered as future and past talk, respectively, and **f** a heatmap of trait optimism superimposed on the self-organized map

The SOMTimeS graphical user interface (GUI) offers important methodological advantages for conversation scientists to directly evaluate the potential number and meaningfulness of clusters. SOMTimeS maps the temporal reference time series for each conversation onto a standard SOM two-dimensional graph (Fig. 12a), thus allowing human analysts to “see” multi-dimensional relationships in two-dimensional space. Because the absolute graphical distance between observations is not a direct measure of temporal reference arc similarity, adding a U-matrix (Fig. 12b) offers “topographical” boundaries marking prominent differences in time-series patterns. When considering a two-cluster solution (Fig. 12c), we observe that the peripheral region of the toroidal SOM mesh is characterized by one cluster; while a second cluster is observed in the central region. This GUI offers analysts visually intuitive access to evaluate the observations in two important and complementary ways. The first uses qualitative methods, such as those used in linguistic and anthropological sciences, to systematically sample conversations from seemingly discrete “geographic regions” of the map to explore whether and how conversation dynamics (e.g., turn-taking etiquette, power dynamics of voice and topics, empathic expressions) indicate clinically meaningful sub-types of interactions.

The second advantage of the SOMTimeS GUI is the ability to overlay onto the same map information about each observation that was not used for clustering, but that may be conceptually relevant to evaluating the clinical relevance of the SOM regions. For example, other work identifies that an important personality trait - the tendency for how optimistically or pessimistically seriously ill people react to uncertainty—has important clinical implications for the process and outcomes of how clinicians, patients and families discuss the future (Robinson et al. 2008; Ingersoll et al. 2019). At the time of study enrollment, patient participants in the PCCRI self-reported their degree of trait optimism on an ordinal scale from high to low. Figure 12f shows a heatmap of trait optimism scores that visually indicate a potentially strong association with regions of the SOM. When considering the two-cluster solution, we observe that the peripheral region is characterized by relatively higher optimism and more future-oriented temporal reference “story arcs” (Fig. 12d) versus a central region with lower optimism and less future-orientated shapes of conversations (Fig. 12e). When considered statistically, trait optimism is significantly associated with peripheral versus central cluster assignment (chi square $p<0.05$).

We propose that the scalability of SOMTimeS for efficiently evaluating time-series phenomena in dynamic clinical conversations, such as story arcs, and the intuitive GUI offers an exceptional opportunity for multi-method research focused on discovering and evaluating clinically meaningful types of healthcare communication.

## Discussion

We present SOMTimeS as a clustering algorithm for time series that exploits the competitive learning of the Kohonen self-organizing map, a pruning strategy and the distance bounds of DTW to improve execution time. SOMTimeS contrasts with other DTW-based clustering algorithms in both its ability to both reduce the dimensionality of, and visualize input features associated with clustering temporal data. We also implemented a similar DTW-distance pruning strategy in K-means for the first time to demonstrate performance gains achieved for what is likely the most popular clustering algorithm to date. The resulting algorithm, K-TimeS, is faster than SOMTimeS because it requires fewer iterations through the data. In terms of accuracy, SOMTimeS has higher assessment indices compared to TADPole, and while the assessment indices are statistically similar with K-TimeS, the additional functionality of the SOM comes with a higher computational cost.

The benchmark experiments in this work are intended to put SOMTimeS in context with state-of-the-art time series clustering algorithms. Keeping the study objectives in mind, execution times are used to demonstrate scalability, and highlight the feasibility of analyzing large time series datasets using SOMTimeS. K-means is perhaps the most popular clustering algorithm and has been proven time and again to outperform state-of-the-art algorithms; however, because of its simplicity, it lacks the interpretability and visualization capabilities of the SOM. TADPole on the other hand, is a state-of-the-art clustering algorithm that organizes data differently from SOMTimeS (and by extension K-TimeS), as evident from the difference in ARI scores (see Fig. 6a), and choice of centroids (i.e., density peaks; see Supplementary Material Section 8.1). For these reasons, the algorithms tested are not direct competitors of one another and each has advantages in their own right.

SOMTimeS learns (i.e., self-organizes) in an iterative manner such that as the number of SOM epochs increase, the execution time per epoch decreases (see Fig. 9b), making higher number of epochs (and thus, corresponding assessment indices) feasible. This reduction in time is also directly proportional to the number of calls to the DTW function at each epoch. The elbow point (at 6 for SOMTimeS with 100 epochs) indicates quick gains in pruning DTW calculations. This same gain is observed when the total number of epochs is set to 10 or 50 (see Supplementary Material Figure S4). SOMTimeS took 40 min to cluster the entire UCR archive using 10 epochs, and less than 300 min when the number of epochs was increased 10-fold. Similarly, the largest dataset in terms of problem size took 5 min to cluster using 10 epochs, and 35 min to cluster at 100 epochs. SOMTimeS demonstrates sub-linear scalability when it comes to increasing the number of epochs. The scalability, fast execution times, and the ease of saving the state (weights) of a SOM make SOMTimeS a potential candidate for an *anytime* algorithm. It possesses the five most desirable properties of anytime algorithms (Zilberstein and Russell 1995; Zhu et al. 2012).

**Concluding remarks** This paper presents a computationally efficient variant of the SOM that uses DTW as a distance measure and a DTW-distance pruning strategy. For comparison purposes, we also implement a similar pruning strategy for K-means, called K-TimeS, and to put its performance in context, we present another state-of-the-art algorithm, TADPole. All three use DTW-distance pruning, and each has their own strengths and weaknesses. They each organize data differently, and the SOM is known for data visualization, dimensionality reduction, and feature selection. For these reasons a direct comparison of the advantages and disadvantages of each algorithm is not possible. SOMTimeS has unique data visualization abilities that require the mesh size to be increased to a value higher then *k*. The pruning strategy presented in this work makes the latter feasible. However, if only classification (or hard clusters) are required, then $K-TimeS$ is the faster and equally accurate clustering algorithm.

## Conclusion and future work

The explosion in volume of time series data has resulted in the availability of large unlabeled time datasets. In this work, we introduce **S**elf-**O**rganizing **M**aps for **t**ime **s**eries (**SOMTimeS**). SOMTimeS is a self-organizing map for clustering and classifying time series data that uses DTW as a distance measure of similarity between time series. To reduce run time and improve scalability, SOMTimeS prunes DTW calculations by using distance bounding during the SOM training phase. This pruning results in a computationally efficient and fast time series clustering algorithm that is linearly scalable with respect to increasing number of observations. SOMTimeS clustered 112 datasets from the UCR time series classification archive in 7 hours with state-of-art accuracy. We also implemented a similar pruning strategy in K-means for the first time to demonstrate performance gains achieved for what is likely the most popular clustering algorithm to date. K-TimeS clustered all the 112 datasets in 6 hours with accuracy scores comparable to SOMTimeS; however, the former lacks the visualization capabilities of the SOM. We applied SOMTimeS to 171 conversations from the PCCRI dataset. The resulting clusters showed two fundamental shapes of conversational stories.

To further improve computational efficiency and clustering accuracy, newer and state-of-the-art variations of SOMs may be used that leverage the same pruning strategy in this work. Improving computational time of DTW-based algorithms is an active area of research, and any improvement in computational speed of DTW can be incorporated in SOMTimeS for the unpruned DTW computations. Finally, SOMTimeS is a uni-variate time series clustering algorithm. To create a multivariate time series clustering algorithm, the pruning strategy will have to be revisited to accommodate the variations of DTW for multi-variate time series. SOMTimeS is a fast and linearly scalable algorithm that recasts DTW as a computationally efficient distance measure for time series data clustering.

### Supplementary Information

Below is the link to the electronic supplementary material.

Supplementary file 1 (pdf 660 KB)
