# Supplementary material for: Somtimes: self organizing maps for time series clustering and its application to serious illness conversations
Source: Data Min Knowl Discov. 2023 Oct 20;38(3):813–39. doi: 10.1007/s10618-023-00979-9 (PMC11069464; doi:10.1007/s10618-023-00979-9)
Supplement: Supplementary file 1 — (pdf 660 KB) [file 10618_2023_979_MOESM1_ESM.pdf]

8 Supplementary Material

This supplementary material provides additional information on the following aspects of the study:

1. Section 8.1 describes the TADPole (Begum et al., 2015) algorithm.

2. Figure S1 show datasets sorted by increasing problem size on arithmetic and log-log scale.

3. Figure S4b shows the percentage of DTW computations by SOMTimeS, K-means, and TADPole for each of the 112 UCR archive datasets.

4. Change in pruning efficiency of SOMTimeS (10 epochs total) as reflected by the calls to DTW function, and execution time over epochs.

8.1 TADPole

TADPole (Begum et al., 2015) is a density based clustering method that uses Density Peaks (Rodriguez and Laio, 2014) as the clustering algorithm and DTW as the distance measure. The Density Peaks algorithm generates cluster centroids (called "density peaks") that are surrounded by neighboring data points that have lower local density and are relatively farther from data points with a higher local density (Rodriguez and Laio, 2014). The algorithm has two phases. It first finds centroids (density peaks), and then assigns data points to the closest centroid. The algorithm requires two input parameters: the number of clusters (k) and the local neighborhood distance d (wherein the local density of a data point is calculated). In this work, when TADPole is used, k is assumed to be known, and the value of d is determined as the distance wherein the average number of neighbors is 1 to 2% of the total number of observations in the dataset, following a rule of thumb proposed by the original authors (Rodriguez and Laio, 2014). TADPole uses upper bound (Euclidean distance) and lower bound (LB_Keogh) to prune unnecessary DTW calculations in the first phase to speed up the clustering. The algorithm has a complexity of O(n^2) where n is the number of time series observations in the input.

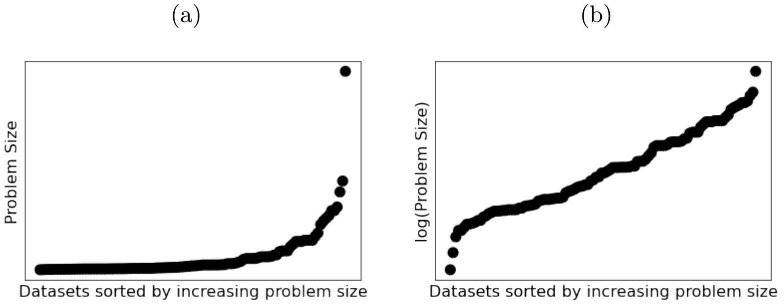

Figure S1: Distribution of all 128 datasets in the UCR archive in terms of (a) problem size and (b) natural log of problem size.

.

Table S1: Clustering performance shown for SOM with Euclidean distance using six assessment indices. Values represent averages over the 112 datasets in the UCR archive; indices closer to 1 represent better performance.

**Algorithm**	**ARI** [7]		**AMI** [8]		**RI** [9]		**H**[10]		**C**[11]		**FMS**[12]	
	avg	std	avg	std	avg	std	avg	std	avg	std	avg	std
**SOM (Euclidean)** 100-iterations	0.19	0.22	0.25	0.25	0.67	0.18	0.25	0.24	0.45	0.35	0.49	0.20

[7] Adjusted Rand Index (Santos and Embrechts, 2009)

[8] Adjusted Mutual Information (Romano et al., 2016)

[9] Rand Index (Hubert and Arabie, 1985)

[10] Homogeneity (Rosenberg and Hirschberg, 2007)

[11] Completeness (Rosenberg and Hirschberg, 2007)

[12] Fowlkes Mallows index (Fowlkes and Mallows, 1983)

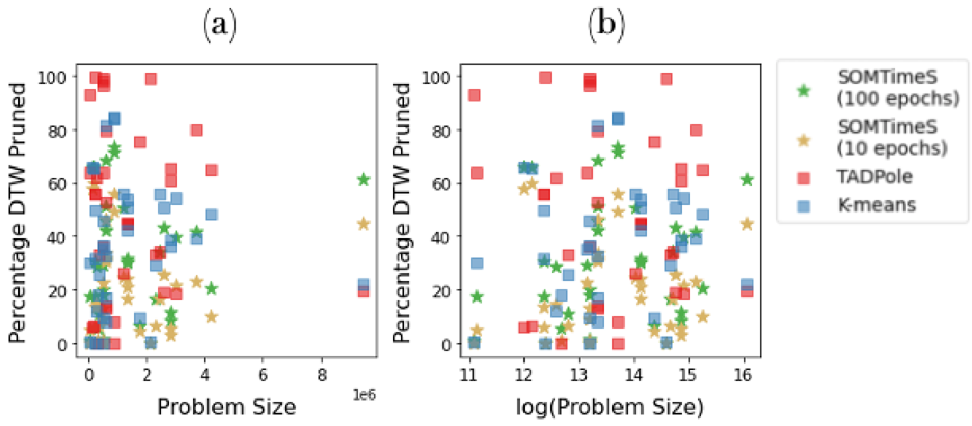

Figure S2: The pruning effect of SOMTimeS (10 epochs shown in blown stars), SOMTimeS (100 epochs in green stars), K-means (blue squares) and TADPole (red squares) measured as the percentage of DTW calls pruned during the clustering of a dataset for varying problem size in (a) linear scale axis and (b) natural log axis.

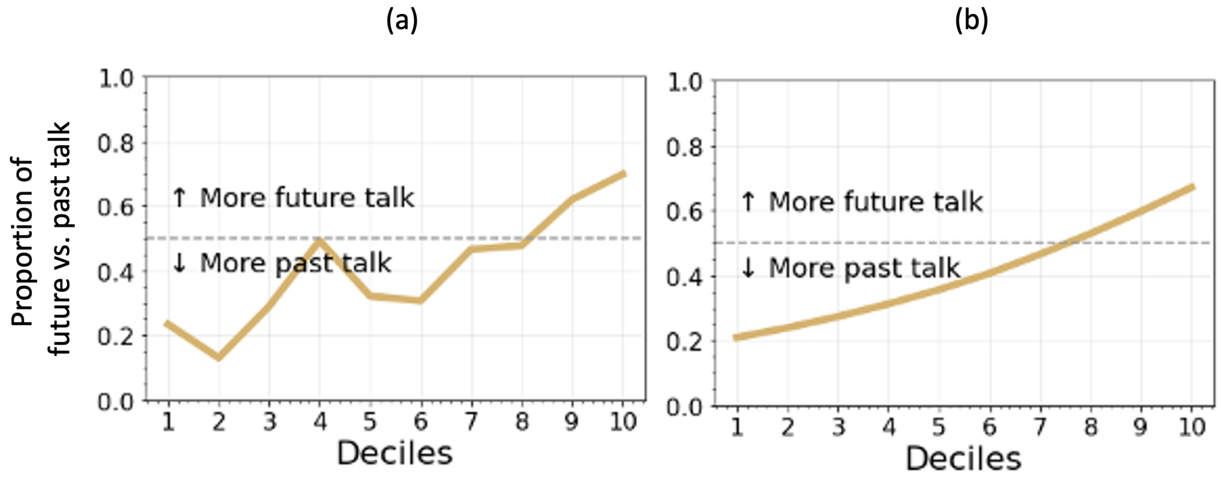

Figure S3: One example of a patient-clinician conversation shown as (a) time series of future versus past talk and (b) a smoothed temporal story arc.

.

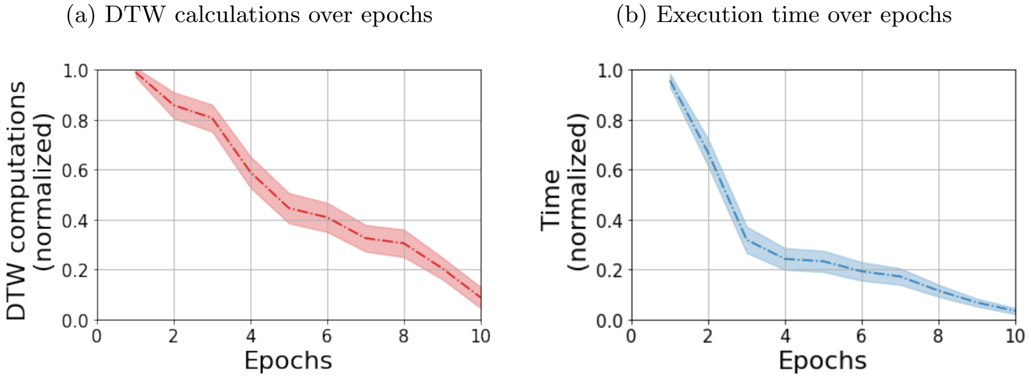

Figure S4: Change in pruning efficiency of SOMTimeS (10 epochs total) as reflected by the calls to DTW function, and execution time over epochs. The dotted line represents the mean value for all datasets after individually normalizing run for each dataset over all epochs. The shaded region corresponds to 95% confidence interval around the mean.
